# Concentric Isokinetic Strengthening Program’s Impact on Knee Biomechanical Parameters, Physical Performance and Quality of Life in Overweight/Obese Women with Chronic Meniscal Lesions

**DOI:** 10.3390/healthcare11142079

**Published:** 2023-07-20

**Authors:** Nadhir Hammami, Amani Mechraoui, Soukaina Hattabi, Pedro Forte, Tatiana Sampaio, Andrew Sortwell, José E. Teixeira, Luís Branquinho, Ricardo Ferraz, Anissa Bouassida

**Affiliations:** 1 Research Unit (UR22JS01) “Sport Sciences, Health and Movement”, High Institute of Sport and Physical Education of Kef, University of Jendouba, Kef 7100, Tunisia; 2 Department of Sports, Higher Institute of Educational Sciences of the Douro, 4560-547 Penafiel, Portugal; luis.branquinho@iscedouro.pt; 3 CI-ISCE, Higher Institute of Educational Sciences of the Douro, 4560-547 Penafiel, Portugal; 4 Research Center in Sports, Health and Human Development, 6201-001 Covilhã, Portugal; tatiana_sampaio30@hotmail.com (T.S.);; 5 Department of Sports, Instituto Politécnico de Bragança, 5300-252 Bragança, Portugal; 6 School of Health Sciences and Physiotherapy, University of Notre Dame Australia, Fremantle, WA 6160, Australia; 7 Department of Sport Sciences, Polytechnic Institute of Guarda, 6300-559 Guarda, Portugal; 8 Department of Sports Sciences, University of Beira Interior, 6201-001 Covilhã, Portugal

**Keywords:** strength dynamometer, meniscus, degenerative meniscal lesion, middle-aged, obesity, continuous mode

## Abstract

Meniscal injuries are one of the most common intra-articular knee injuries. Different treatments are presented depending on the symptoms and duration of meniscus tears, such as arthroscopic partial meniscectomy, physiotherapy, or even pharmacological treatment. The purpose was to investigate the effect of a concentric isokinetic knee muscle strengthening program on strength, joint range of motion, physical performance, quality of life and pain tolerance in overweight/obese women with chronic meniscal lesions. Twenty-four overweight/obese women were randomized into two groups. A control group (CG) performed a usual rehabilitation program plus isokinetic muscle strengthening (IMS) in the continuous passive motion mode and measured with an isokinetic dynamometer. An experimental group (EG) performed the same program in combination with IMS in the isokinetic active mode. The peak torque of the knee extensors (PTE) and flexors (PTF), sit-to-stand test, stair climb test, joint amplitude, heel-to-buttock distance, Thessaly test, KOOS questionnaire of pain and quality of life were measured pre- and post-protocol. After the intervention, the sit–stand number for both groups was significantly higher (*p* < 0.001) and the time to climb stairs was significantly reduced for the EG (*p* < 0.001). A significant improvement in joint range of motion, life quality (*p* < 0.001), relief in knee pain (*p* < 0.001) and in the post-program evaluation at the EG (*p* < 0.05) was noted. There was no significant difference in PTE and PTF between groups at 60°/s and 180°/s. The IMS in the active mode could be an effective therapeutic modality in managing middle-aged patients suffering from a degenerative meniscal tear.

## 1. Introduction

In middle-aged adults, meniscal injuries are one of the most common intra-articular injuries of the knee and are the most frequent cause of surgical interventions performed by orthopedic surgeons [1]. For instance, in the United States alone, meniscus injuries are encountered by 6–8% of active young adults annually, and this number is increased in the elderly population. Although tears are more frequently symptomatic in younger and physically active individuals, they occur more commonly in the elderly, impacting approximately 31% of the aged population [2]. Studies have shown that the distribution of degenerative meniscal lesions between men and women is variable [3,4]. Indeed, the prevalence is high in men aged 70 to 90 years, at about 50%, while in women aged 50 to 60 years, it is around 16% [4].

In individuals with overweight, both a higher prevalence and severity of early degenerative changes in the knee in middle-aged individuals without radiographic osteoarthritis (OA) and significantly increased cartilage lesion progression (of any grade) were noted [5], thereby making the joint more susceptible to injury. Meniscal lesions are typically categorized as traumatic or non-traumatic (i.e., degenerative) according to their etiology. Traumatic meniscal lesions occur predominantly in active young individuals and are often triggered by severe trauma [6]. Degenerative tears are typically seen in middle-aged or older people and often accompany knee osteoarthritis [7].

Depending on the symptoms and duration of meniscus tears, surgeons may opt for different treatments, including arthroscopic partial meniscectomy (APM) or conservative treatments such as physical therapy (PT) or pharmacological treatment [4,8]. For meniscus lesions, conservative treatment is becoming the preferred treatment. Exercise therapy has been indicated as a promising therapeutic option for individuals with knee degeneration [9]. Treatment aims to increase knee function while also reducing joint pain.

Researchers indicated that isokinetic muscle strength assessment could be used to accurately test muscle strength, adjust the nerve control of muscle function, and enhance joint stability, flexibility and movement coordination [10,11]. This makes it among the most valuable tools for analyzing and developing muscle strength and joint range of motion [10]. This is why it has been used as a means of muscle strengthening coupled with neuromuscular electrical stimulation intervention [11] in various pathologies of the knee such as gonarthrosis [12], and patellofemoral syndrome [13,14]. The reliability of the test results, notably for lower limb muscles, has enabled isokinetic dynamometers to be regarded as a standard for measuring muscular strength [15,16]. Indeed, isokinetic dynamometers as muscle strength assessment tools for meniscal lesion studies are well-documented [17,18,19,20,21]. However, patients state that the pain caused by a knee pathology constitutes a limitation to carrying out isokinetic tests, but if tolerated, it is reduced following a muscle strengthening program (e.g., the case of knee osteoarthritis) [11]. Furthermore, isokinetic movements offer contractions modes via mechanical functioning, which help to produce a tolerated isokinetic work [22]. This is the continuous passive motion (CPM) in addition to the isokinetic active motion (AM) mode initially produced by the dynamometer [23,24]. The mode of CPM is generally used in OA, causing pain such as gonarthrosis [11,12,24] and neurological diseases [22,25], which makes it very suitable and useful for muscle evaluation strengthening in degenerative meniscus lesions (DMLs).

Since performing APM in all patients with knee pain and a meniscal tear is inappropriate, surgical treatment should not be considered the first-line intervention [26]. Consequently, it is essential to establish a more appropriate conservative regimen that can be attributed to any non-surgical intervention, such as immobilization of the knee with a splint, using English canes, oral analgesics, cryotherapy and physiotherapy. Hence, a first-line treatment diversifies the management types to give the physician and patient more therapeutic options. Therefore, this study investigates the effect of an isokinetic muscle strengthening program on knee biomechanical parameters, physical performance and quality of life in overweight/obese women as a therapeutic approach for middle-aged patients with degenerative meniscus lesions, DMLs.

## 2. Materials and Methods

### 2.1. Participants

The participants’ recruitment was carried out for six months. For study eligibility, they had to be female, aged between 35 and 55 years, and suffer from knee pain and discomfort for the majority of the previous 30 days prior to the study (one and/or both affected knees), with a body mass index (BMI) above 26 kg/m^2^ and a radiological diagnosis indicating stage 2 degenerative meniscal lesions. The meniscal lesion stages and leg dominance were identified by the same physical doctor. Patients were assessed for a clinical determination of footedness to identify their preferential side (if they were right- or left-footed) in accordance with tasks identified by Schneiders et al. [27]. The preferential side was considered to be the dominant side/leg. The affected knee was always considered the dominant side/leg regardless of its designation as the patient’s preferential or non-preferential side. If both knees were altered, the preferential side was considered the dominant side/leg.

Furthermore, patients undergoing meniscal surgery, with health problems, heart diseases, and cancer under treatment, pregnant patients and unavailable patients were ruled out. In addition, exclusion criteria concerned withdrawal from the study, absence in 2 consecutive sessions in a week, injury, and a declaration of demotivation or non-implication in carrying out the protocol. G-Power software (G*Power 3.1.9.6) was used to compute the sample size required. An effect size of f = 0.40, an actual power of 0.85, and α = 0.05 were included in analysis. A minimum sample size of 16 participants was required hence the selection of 24 participants that also met the criteria [28]. The risks and advantages connected with the study’s experimental techniques were explained orally and in writing. All participants who wished to participate in the study signed an informed consent form and met the eligibility criteria. The study followed the Helsinki Declaration for human experimentation, and the protocol was entirely approved by the Ethical and Scientific Committees of the “High Institute of Sport and Physical Education of Kef, University of Jendouba” Research Unit.

**Figure 1:**
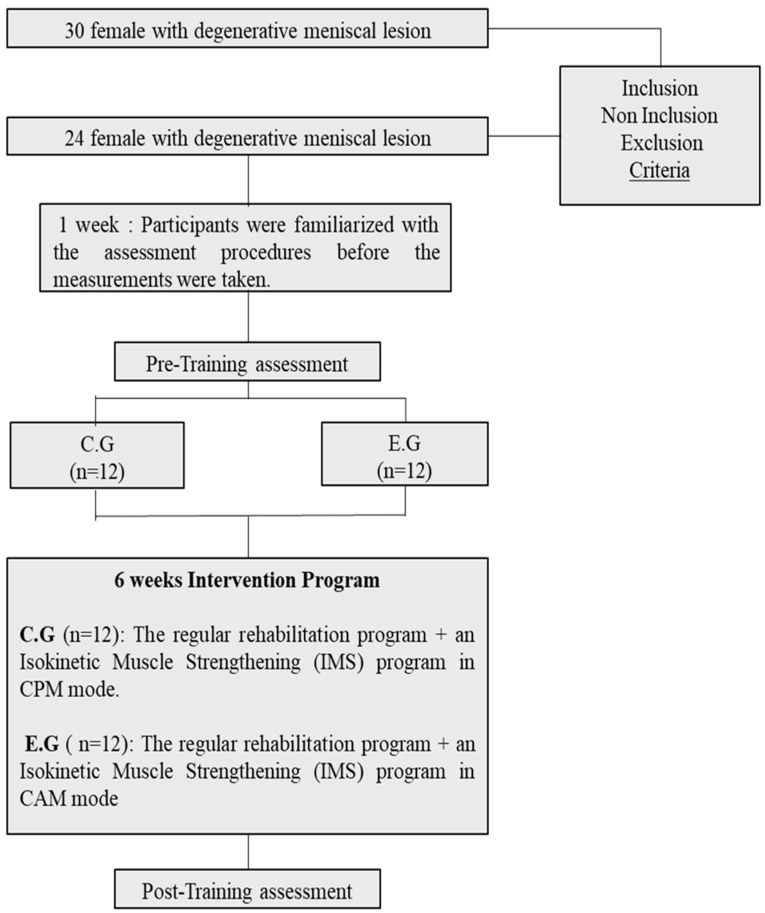
Study Design Chart.

This study was randomized controlled and following initial testing, our study population was randomized into two groups. Randomization and allocation to groups were conducted using a random number generator based on the type of procedure undergone (Figure 1). The control group (CG) performed a regular rehabilitation program performed twice a week containing a variety of physical exercises for muscle strengthening, joint mobilization, agility and stretching followed by an isokinetic muscle strengthening (IMS) program in the continuous passive motion (CPM) mode, in which the knee moves through the range of motion in concentric mode with machine assistance. The regular program lasted approximately 60 to 90 min for each session. Each session included 20 min of warm-up, 35 min of strengthening and neuromuscular exercises and 5 min of cool down (active recovery). All the exercises were carried out at the rate of 3 sets of 10 repetitions in the first week, and then an increase in the additional load or surface change of each exercise was effected, during week 2 to week 12. The rest period was 30 s between sets for the first four weeks, and 30 s to 1 min from the fifth week [20]. The recovery time between exercises was 3 min throughout the 12 weeks. Furthermore, based on the study of [29], ten exercises were included in this program and were performed in the following order: squat, single-leg squat, step-up, knee stability in pull loop, hamstring on fitball, single-leg knee extension, skating, limping cross, heel raises and heel dig bridging. The program adhered to the principles of load training progression for beginners and intermediate middle-aged people. The experimental group (EG) performed the same regular rehabilitation program and an IMS program in the active motion (AM) mode by using maximum intensity concentric contractions of knee extensors and flexors (at 60°/s and 180°/s angular speeds, respectively), with an articular amplitude of 80° [17].

The following training program was performed for all groups [30]:

- Briefly, there were r12 sessions over 6 weeks at a rate of two sessions per week, with 2 rest days between two sessions (Monday and Thursday for some patients and Tuesday and Friday for other patients).
- After a 10 min warm-up on an ergocycle, dynamic stretching and static stretching of the quadriceps, hamstrings, adductors, gluteal and calf muscles were performed (10 min).
- Then, IMS in the concentric mode was conducted, with the first session consisting of 1 set of 5 maximum knee flexion/extension repetitions for each knee. Overall, the dominant leg was always trained first on the isokinetic machine.
- The number of sets increased progressively with the addition of 1 set per session (+1 set/session), to reach 12 sets of five repetitions at the 12th session with 1 min 30 s to 2 min of recovery between sets from the second week.
- Tolerance was monitored at each session and effectiveness was determined at the end of the program by performing a maximal isokinetic evaluation of knee flexors and extensors.

### 2.2. Procedures

General anthropometric data were measured barefoot with as little clothing as possible. Height was tested with a non-deformable measuring scale on the floor, with feet together and looking forwards. BMI, body weight, and percentage of fat were measured with an impedance meter (OMRON BF-212, Kyoto, Japan). Patients were familiarized with the test procedures before taking the measurements. Prior to testing, participants performed a standard warm-up consisting of 10 min of pedaling on an ergocycle (Care Ergo V, Paris, France) with the number of rounds per minute being between 70 to 90, and a heart rate not exceeding 140 beats per minute, followed by muscle stretching. The test durations varied between 1 h 45 min and 2 h 30 min. The testing order was randomized for participants’ motivation and to avoid tampering with the results.

#### 2.2.1. Isokinetic Evaluation

**Figure 2:**
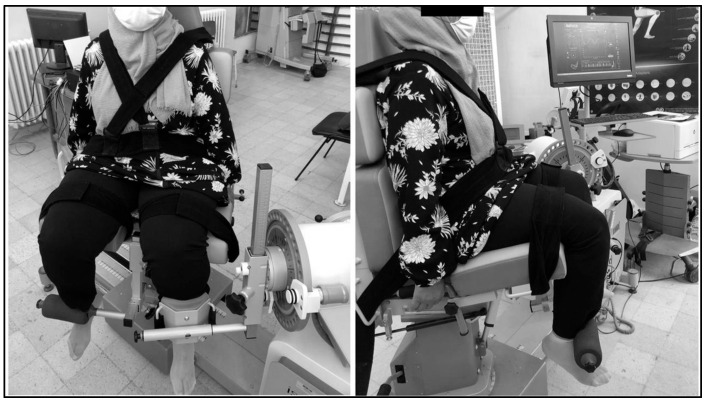
Placement of the patient on the isokinetic dynamometer.

We used an isokinetic dynamometer of the isoforce type (TUR GmbH, Germany) to assess muscle strength. The subject was instructed to be seated with their back at 90° with an adjustment seat for thigh length during examinations (Figure 2). The rotation center of the knee joint, namely the lateral epicondyle of the femur, was visually aligned with the dynamometer axis’ laser projection. With a resistive padded counter-bearing placed downstream of the leg, and three fingers from the lateral malleolus without disrupting the ankle’s range of motion, the assessed limb was firmly attached to the lever arm of the dynamometer. The thigh, pelvic girdle, and trunk were tightly strapped to avoid compensation. The patient had to perform maximal knee flexions/extensions in the concentric mode at two angular speeds, 60°/s and 180°/s for each leg, always starting with the dominant side/leg. A specified warm-up was carried out beforehand for the patient to become comfortable with the activity after correcting the gravity torque and adjusting the mechanical stops of the dynamometer adapted for a “Range of Motion” (ROM) of 80° [11,31]. Each test consisted of five submaximal warm-up contractions followed by five maximum evaluation contractions, with a 30s rest time between the warm-up and the assessment. The recovery time between the two angular velocity tests was 4 min. The isokinetic parameter retained for the analyses was the peak torque related to body weight. During testing, the investigator provided vocal instruction and directions.

#### 2.2.2. Physical Performance Evaluation

For the sit-to-stand test, a chair with a hard seat whose floor-to-seat height was 45 cm was stabilized by placing it against a wall. The test began with the participant seated in the middle of the chair, with their back straight, and feet approximately shoulder-width apart and placed on the floor at an angle slightly back from the knees, with one foot slightly in front of the other to help maintain balance when standing. The participant was asked to sit with their upper limbs folded across their chest and then to stand up all the way and sit down again without using her arms as quickly as possible [32]. The score was the total number of stands executed correctly within 30 s. Incorrectly executed stands were not counted [33]. Participants completed two trials, each separated by a 2 min rest period. The mean score was used for analysis.

The stair climbing test consisted of a measure of the time required for participants to climb eleven stairs at the height of 20 cm. It is a valid and reliable test with an Interclass correlation coefficient of 0.94 and good inter-rater reliability [34]. Participants were instructed to climb as fast as possible without jumping [35]. They completed two trials with 2 min of rest. The mean score was retained for analysis. The time required to climb eleven steps was measured with a stopwatch with an accuracy of 1/100th of a second.

#### 2.2.3. Clinical Exam

**Figure 3:**
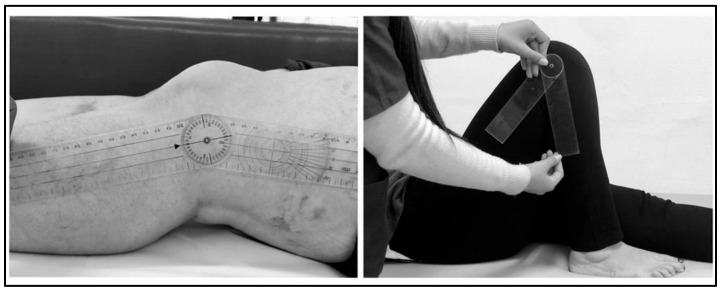
Joint amplitude measurement.

Knee flexion and extension (amplitude) joint ranges were measured using a goniometer by centering it on the knee’s lateral condyle and aligning one arm of the goniometer along the femur axis toward the greater trochanter and the other along the leg toward the lateral malleolus in the supine position [11,36] (Figure 3).

**Figure 4:**
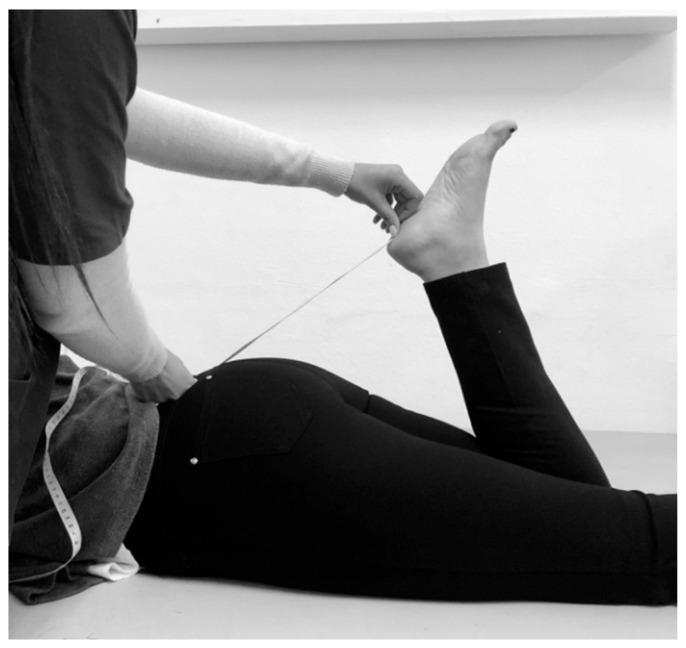
Heel-to-buttock distance (HBD) measurement.

Heel-to-buttock distance (HBD) was measured to detect potential loss of muscle size or reduced flexibility, primarily of the quadriceps [37]. The examiner passively bended their knee and used a meter tape, to measure the HBD increase in cm (Figure 4).

The Thessaly test was originally described by Karachalios et al. [25]. It attempts to reproduce dynamic load transmission in a knee joint. The examiner supports the patient by holding out their outstretched hands. The patient then rotates his knee and body both internally and externally three times, keeping the knee in slight flexion at 5°. The same testing procedure is then repeated with the patient maintaining the knee in greater flexion at 20°. Positive test results in joint line discomfort, locking, or catching refer to the slightly flexed knee with rotation. In order to improve the test findings, pain was measured using the Arabic Numeric Pain Rating Scale (ANPRS) which was derived from the NRS English version, translated and validated by [38,39].

All clinical tests were conducted by the same physical doctor and they were performed twice, once during the test familiarization week and the other during the pre-training assessment period.

#### 2.2.4. Evaluation of Knee-Related Quality of Life and Pain

The Knee Injury and Osteoarthritis Outcome Score (KOOS) Arabic version questionnaire was used [40]. The subscores for knee-related quality of life (KOOS-QOL) and pain (KOOS-Pain) were retained for this section.

### 2.3. Statistical Analysis

The STATISTICA 10.0 software (StatSoft, Inc. Tulsa, OK, USA) for Windows was used for analysis. The findings are reported as mean values ± standard deviation. The normality of the distribution of all variables was checked with the “Shapiro-Wilk” test to determine the selection of tests (parametric or non-parametric). A two-way repeated measures analysis of variance, ANOVA (time × groups, respectively, with dependent and independent variables), was conducted. A comparison by pair was performed using the Bonferroni/LSD post hoc test. The effect size (*η_p_*^2^) was examined to investigate the size of the difference between the variables (classified as 0.01 = small, 0.06 = medium, 0.14 = large). The level of statistical significance was considered to be at *p* < 0.05.

## 3. Results

### 3.1. Isokinetic Muscle Strength

As a result of conducting the ANOVA test, the knee PTE comparison between pre- and post-program groups at 60°/s showed non-significant group (F_(1.22)_ = 0.634; *p* = 0.434; *η_p_*^2^ = 0.028), pre–post-program (F_(1.22)_ = 0.019; *p* = 0.892; *η_p_*^2^= 0.0008), group × pre–post-program interaction (F_(1.22)_ = 0.719; *p* = 0.405; *η_p_*^2^ = 0.032), group × RL interaction (F_(1.22)_ = 0.187; *p* = 0.669; η_p_^2^ = 0.008), and RL × pre–post-program interaction (F_(1.22)_ = 0.087; *p* = 0.771; *η_p_*^2^ = 0.004) effects, but a significant effect of RL (F_(1.22)_ = 10.167; *p* = 0.004; *η_p_*^2^ = 0.316) and pre–post-program × RL × group interaction (F_(1.22)_ = 6.743; *p* = 0.016; *η_p_*^2^ = 0.235) was observed. The PTEs of the right knee were significantly improved after the program compared to those before for the CG (*p* = 0.0147). Concerning the left knee, results showed a significant improvement in the post-program evaluation for the EG compared to that in the CG (*p* = 0.003) (Table 1).

In addition, the knee PTF comparison between the pre- and post-program groups at 60°/s showed non-significant group (F_(1.22)_ = 1.251; *p* = 0.275; *η_p_*^2^ = 0.054), pre–post-program (F_(1.22)_ = 0.505; *p* = 0.485; *η_p_*^2^ = 0.022), group × pre–post-program interaction (F_(1.22)_ = 0.089; *p* = 0.768; *η_p_*^2^ = 0.004), group × RL interaction (F_(1.22)_ = 1.032; *p* = 0.321; *η_p_*^2^ = 0.045), RL × pre–post-program interaction (F_(1.22)_ = 0.526; *p* = 0.476; *η_p_*^2^ = 0.023) and RL (F_(1.22)_ = 3.664; *p* = 0.069; *η_p_*^2^ = 0.143) effects, but a significant effect of pre–post-program × RL × group interaction (F_(1.22)_ = 5.590; *p* = 0.0273; *η_p_*^2^ = 0.203) was found. Furthermore, the left knee PTFs were significantly improved after the program compared to those before for the CG (*p* = 0.028), and they were significantly different between the EG and the CG prior to the program (*p* = 0.019) (Table 1).

For the speed of 180°/s, the ANOVA comparison of knee PTEs between the pre- and post-program groups showed non-significant group (F_(1.22)_ = 0.140; *p* = 0.711; *η_p_*^2^ = 0.006), group × pre–post-program interaction (F_(1.22)_ = 0.599; *p* = 0.447; *η_p_*^2^ = 0.027), group × RL interaction (F_(1.22)_ = 3.483; *p* = 0.075; *η_p_*^2^ = 0.137), RL × pre–post-program interaction (F_(1.22)_ = 2.244; *p* = 0.148; *η_p_*^2^ = 0.093) and pre–post-program × RL × group interaction (F_(1.22)_ = 1.037; *p* = 0.319; *η_p_*^2^ = 0.045) effects, but a significant effect of the pre–post-program (F_(1.22)_ = 7.028; *p* = 0.015; *η_p_*^2^ = 0.242) and RL (F_(1.22)_ = 18.550; *p* = 0.0002; *η_p_*^2^ = 0.457) was observed. Moreover, a significant improvement of the right (*p* = 0.0003) and the left (*p* = 0.00095) knee PTEs were shown after the program for the CG, while the right knee PTEs were significantly different from those of the left knee measured after the program (*p* = 0.03). Similarly, the EG right knee PTEs after the program were significantly different compared to the left knee PTEs prior to the program (*p* = 0.008) (Table 1).

Otherwise, the comparison via ANOVA of knee PTFs between pre- and post-program groups at 180°/s showed non-significant group (F_(1.22_) = 0.033; *p* = 0.857; *η_p_*^2^ = 0.002), RL × pre–post-program interaction (F_(1.22)_ = 3.489; *p* = 0.075; *η_p_*^2^ = 0.137), pre–post-program × RL × group interaction (F_(1.22)_ = 1.634; *p* = 0.214; *η_p_*^2^ = 0.069) effects. However, significant pre–post-program (F_(1.22)_ = 11.056; *p* = 0.003; *η_p_*^2^ = 0.334), group × pre–post-program interaction (F_(1.22)_ = 5.098; *p* = 0.034; *η_p_*^2^ = 0.188), group × RL interaction (F_(1.22)_ = 4.533; *p* = 0.045; *η_p_*^2^ = 0.17) and RL (F_(1.22)_ = 19.718; *p* = 0.0002; *η_p_*^2^ = 0.473) effects were found. The right and left knee PTFs were significantly improved after the program compared to those before for the CG (respectively *p* = 0.001, *p* = 0.002) with a significant difference between the EG pre-program and the CG after the program (*p* = 0.006). For the CG, the right knee PTFs were significantly higher than the left knee PTEs after the program (*p* = 0.00007) (Table 1).

### 3.2. Physical Performance Evaluation

#### 3.2.1. Sit-to-Stand Test

The highly significant-difference group (F_(1.22)_ = 12.492; *p* < 0.001; *η_p_*^2^ = 0.362), pre–post-program (F_(1.22)_ = 143.690; *p* < 0.001; *η_p_*^2^ = 0.867) and group × pre–post-program interaction (F_(1.22)_ = 17.935; *p* < 0.001; *η_p_*^2^ = 0.449) was determined. Moreover, the sit–stand number was significantly higher post than that pre-program for both groups (*p* < 0.001). A highly significant improvement was registered in the post-program assessment in the EG (*p* < 0.001) compared to the CG (Table 2).

#### 3.2.2. Stair Climbing Test

The highly significant difference group (F_(1.22)_ = 5.109; *p* < 0.05; *η_p_*^2^ = 0.188), pre–post-program (F_(1.22)_ = 50.222; *p* < 0.001; *η_p_*^2^ = 0.695) and group × pre–post-program interaction (F_(1.22)_ = 15.355; *p* < 0.001; *η_p_*^2^ = 0.411) was noted. The time required to climb stairs was significantly reduced after the intervention compared to that before for the EG (*p* < 0.001). There was also a significant improvement in the post-program evaluation of the EG compared to that of the CG (*p* < 0.05) (Table 2).

### 3.3. Clinical Exam

#### 3.3.1. Knee Amplitude Test

In terms of flexion to the right, a non-significant effect of the group (F_(1.22)_ = 0.400; *p* = 0.534; *η_p_*^2^ = 0.018) and group × pre–post-program interaction (F_(1.22)_ = 0.353; *p* = 0.558; *η_p_*^2^ = 0.016) but a significant pre–post program (F_(1.22)_ = 77.172; *p* < 0.001; *η_p_*^2^ = 0.778) effect was observed. For both groups, the joint ranges of knee flexion to the right were significantly improved after the intervention compared to those before (*p* < 0.001). There was a non-significant improvement in the post-program evaluation for the EG compared to that for the CG (*p* > 0.05) (Table 3).

In terms of flexion to the left, there was a non-significant effect of the group (F_(1.22)_ = 0.521; *p* = 0.478; *η_p_*^2^ = 0.023) and group × pre–post-program interaction (F_(1.22)_ = 0.873; *p* = 0.360; *η_p_*^2^ = 0.038) but a significant pre–post-program effect (F_(1.22)_ = 65.409; *p* < 0.001; *η_p_*^2^ = 0.748). A significant improvement reflected by an increase in the joint ranges of knee flexion to the left after the intervention for the two groups (*p* < 0.001) was found but there was no significant improvement between groups post-program (*p* > 0.05) (Table 3).

In terms of extension to the right, a non-significant effect of the group (F_(1.22)_ = 0.486; *p* = 0.493; *η_p_*^2^ = 0.022) and group × pre–post-program interaction (F_(1.22)_ < 0.001; *p* = 1.000; η_p_^2^ < 0.001) but a significant pre–post-program (F_(1.22)_ = 13.538; *p* = 0.001; *η_p_*^2^ = 0.381) effect was observed. The amplitude of the joint ranges of knee extension to the right did not improve for either group after the program or between groups (*p* > 0.05) (Table 3).

In terms of extension to the left, a non-significant effect of the group (F_(1.22)_ = 1.081; *p* = 0.309; *η_p_*^2^ = 0.047) and group × pre–post-program interaction (F_(1.22)_ = 2.099; *p* = 0.161; *η_p_*^2^ = 0.087) but a significant effect of the pre–post-program (F_(1.22)_ = 14.191; *p* = 0.001; *η_p_*^2^ = 0.392) was noted. The joint ranges of knee extension to the left were significantly improved after the intervention for the CG (*p* < 0.001) (Table 3).

#### 3.3.2. Heel-to-Buttock Distance

There was a non-significant effect of the group (F_(1.22)_ = 0.002; *p* = 0.961; *η_p_*^2^= 0.0001) pre–post-program (F_(1.22)_ = 1.683; *p* = 0.208; *η_p_*^2^ = 0.071), group × pre–post-program interaction (F_(1.22)_ = 0.034; *p* = 0.855; *η_p_*^2^ = 0.001), RL × group interaction (F_(1.22)_ = 1.030; *p* = 0.321; *η_p_*^2^ = 0.045), pre–post-program × RL interaction (F_(1.22)_ = 1.678; *p* = 0.209; *η_p_*^2^ = 0.071) and pre–post-program × RL × group interaction (F_(1.22)_ = 1.678; *p* = 0.209; *η_p_*^2^ = 0.071) (Table 4).

#### 3.3.3. Pain Assessment with Thessaly Test

The results indicated a non-significant effect of the group (F_(1.22)_ = 2.654; *p* = 0.118; *η_p_*^2^ = 0.118) pre–post-program (F_(1.22)_ = 0.343; *p* = 0.564; *η_p_*^2^ = 0.015), group × pre–post-program interaction (F_(1.22)_ = 0.021; *p* = 0.885; *η_p_*^2^ = 0.0009), RL × group interaction (F_(1.22)_ = 0.328; *p* = 0.572; *η_p_*^2^ = 0.015) and pre–post-program × RL × group interaction (F_(1.22)_ = 0.328; *p* = 0.572; *η_p_*^2^ = 0.015), but a significant effect of the RL (F_(1.22)_ = 257.433; *p* < 0.001; *η_p_*^2^ = 0.921) and pre–post-program × RL interaction (F_(1.22)_ = 5.254; *p* = 0.032; *η_p_*^2^ = 0.193). Pain sensation was significantly reduced after the intervention for the EG and CG (*p* < 0.001) when externally rotating their knees (to the left). In addition, it was noted that there was a significant difference between the EG pre-program while internally rotating (to the right) and the post-program while externally rotating (to the left) (*p* < 0.05). Similarly, there was a significant difference between the EG prior to the program while internally rotating and the CG post-program while externally rotating (*p* < 0.001; d = 1.974), as well as a significant difference between the EG prior to the program while externally rotating and the CG post-program while internally rotating (*p* < 0.001) (Table 4).

### 3.4. Quality of Life and Pain Assessment

A significant quality of life group effect (F_(1.22)_ = 4.479; *p* = 0.046; *η_p_*^2^ = 0.169) with a significant training effect (F_(1.22)_ = 179.279; *p* < 0.001; *η_p_*^2^ = 0.890) and a non-significant effect of the group × training interaction (F_(1.22)_ = 0.743; *p* = 0.398; *η_p_*^2^ = 0.033) was recorded. Indeed, the quality of living was significantly improved after the intervention for both groups (*p* < 0.001), but did not improve significantly between groups after the program (*p* > 0.05) (Table 5).

Moreover, a non-significant pain group effect (F_(1.22)_ = 2.523; *p* = 0.126; *η_p_*^2^ = 0.103), of the group × training interaction (F_(1.22)_ = 0.944; *p* = 0.341; *η_p_*^2^ = 0.041) and a significant training effect (F_(1.22)_ = 152.378; *p* < 0.001; *η_p_*^2^ = 0.874) were reported. The pain sensation was significantly reduced after the program for both groups compared to that before (*p* < 0.001) but did not change between groups in the post-program (*p* > 0.05) (Table 5).

## 4. Discussion

Collectively, meniscal injuries represent one of the most common intra-articular knee injuries and are one of the most frequent causes of surgical interventions performed by orthopedic surgeons. As far as is known, no previous study has attempted to use an isokinetic muscle strengthening program as a therapeutic approach for middle-aged patients with a degenerative meniscus lesion. Thus, the study’s purpose was to investigate the effect of a concentric isokinetic strengthening training program on knee muscle strength and flexibility, range of motion, motor performance, quality of life and pain tolerance in overweight or obese women with chronic meniscal lesions. We attempted to use an IMS program as a therapeutic approach for middle-aged patients with a degenerative meniscus lesion. As conservative treatment is becoming the preferred treatment, several randomized controlled clinical trials and the ESSKA Meniscus Consensus Project recommend against APM as the first-line treatment for managing knee pain in patients affected by DML with no radiographic knee osteoarthritis [4,41].

With regard to physical performance tests, the number of sit–stands performed during the 30 s for both groups was significantly higher after the intervention than that before. Correspondingly, a highly significant improvement was registered in the post-program assessment in the EG compared to in the CG. Furthermore, the time required to climb stairs was significantly reduced after the intervention compared to that before for the EG. There was a significant improvement in the post-program evaluation in the EG (*p* < 0.05) compared to that in the CG. Like any modality of muscular strengthening, the adequate elaboration of the training load induced by the isokinetic dynamometer causes an increase in the pathological knee muscle strength [42]. The muscular development observed would be responsible for the improvement in performance assessed through physical tests. Hence, the results obtained in this study highlight the benefits of the IMS program on physical performance. As the management of symptomatic meniscal tear transitions to conservative approaches, our results also highlight the importance of appropriately strengthening the quadriceps’ and hamstrings’ musculature. These findings are similar to the results of Luc-Harkey et al. [43], who showed that greater muscle strength in the quadriceps and hamstrings was associated with less pain and difficulty in performing activities of daily living and better mobility in individuals with osteoarthritis and symptomatic meniscal tear. Concretely, the IMS program induced an improvement in physical performance for the EG implying its worthiness of recommendation for the better management of patients with DML.

In addition, the results show that the pain sensation was significantly reduced after the intervention than before for both groups when externally rotating their knee (to the left) while conducting the Thessaly test. In addition, the joint ranges of knee flexion to the right/left significantly improved after the intervention for both groups. Accordingly, these findings suggest an improvement in knee function and relief in knee pain. They could be explained by the advantages of resistance training which increases the strength and volume of the motor muscles of the knee joint, reduces its friction, and decreases the biomechanical stresses due to the weight of the body upper part via effect depreciation [44]. Previous studies found similar outcomes [45,46,47]. Yim et al. [47] found no significant differences between arthroscopic meniscectomy and nonoperative management with strengthening exercises in terms of knee pain relief and improved knee function in patients after 2 years of follow-up. Another study demonstrated no significant differences in clinical outcomes, such as relief of knee pain or improved knee function, between patients who underwent arthroscopic meniscal surgery and those who underwent conservative management for degenerative meniscal tear [46]. Therefore, the efficacy of arthroscopic surgery was not superior to that of conservative management in this type of patient. The surgical approach can be considered in the case of a negative response following conservative treatment [41,48]. There may, however, be a small-to-moderate benefit from APM compared to physiotherapy for patients without osteoarthritis [26]. However, overall, the results obtained in our study highlight the benefits of conservative management, particularly with the IMS program being proposed as a first choice of treatment for degenerative meniscal tears, with an improvement in clinical test results in both groups induced through two isokinetic interventions, namely in the CPM and AM modes.

Moreover, knee muscle weakness is thought to be among the earliest and most significant indicators in patients with knee osteoarthritis [49]. Hence, it is plausible that strength deficits, especially in the quadriceps, may also occur in degenerative meniscal tear cohorts, a group known to have an increased risk of developing OA. In this context, isokinetic dynamometry is one of the most reliable and widespread approaches to assessing muscle strength [16]. Using this approach, we found no significant difference in concentric knee flexor or extensor (body weight adjusted) strength between groups at a velocity of 60°/s and 180°/s which can be explained by the rather short duration of the intervention program (6 weeks). However, we noted an improvement in isokinetic quadricep strength for both programs.

Similarly, compared with APM-treated cases, a 12-week supervised neuromuscular and strength exercise therapy regimen demonstrated clinically applicable and statistically relevant improvements in isokinetic quadricep muscle strength immediately after program completion. Surgery was not associated with changes in muscle strength three months post-surgery, and exercise seems essential to improving muscle strength in these patients. Practitioners can investigate using this valuable therapeutic exercise regimen in the management of middle-aged patients suffering from a degenerative meniscal tear to increase knee muscle strength and improve functional performance [20]. In this context, a different supervised exercise therapy demonstrated favorable outcomes in improving thigh muscle strength, at least in the short term, compared to surgery [9]. Thus, it can be assumed that the IMS program can benefit this population as a therapeutic modality. However, a longer intervention duration is suggested. Furthermore, the isokinetic evaluation methodology used in our work was standardized, with consistency in the warm-up, dynamometer position, stabilization, gravity correction, contraction modes, angular contraction, angular velocities, repetitions, recovery, information and encouragement. However, the isokinetic modes chosen for CPM and AM constitute a further alternative. Indeed, for CPM, the dynamometer moves the leg isokinetically, and every force, however small, applied by the patient is adapted and recorded over the chosen range of movement [23]. At the AM level, the patient must provide the effort unlike in the CPM mode. This remains a controlled effort [50]. In both cases, the IMS appears to improve knee muscle strength, although a longer training duration is also recommended. The resistance imposed by the isokinetic machine seems to be the reason. Indeed, the dynamometer induces an additional load which still trains the knee muscles regardless of the mode administered (CPM or AM) with an advantage for the latter.

The study found that patients in both groups had reduced pain with a significant improvement in “quality of life”. Similarly, Herrlin et al. [45] found that arthroscopic partial meniscectomy (APM) followed by supervised exercise was not superior to supervised exercise alone in terms of reduced knee pain, and improved quality of life. Moreover, Østerås et al. [51] reported that pain scores and quality of life scores were enhanced in 36 sessions over a 3-month time-frame. It seems that an increase in muscle strength, even if minimal, helps to reduce the pressure on the knee, thus decreasing the pain sensation and the moving fear. This would have an impact on mood which would impact quality of life [11]. In contrast, a systematic review and meta-analysis identified a small but statistically significant effect in favor of APM compared to physical therapy (PT) in terms of pain scores up to the 12-month follow-up point. Nevertheless, MPA and PT yielded comparable outcomes at the 24-month follow-up point [52]. Since then, increased knee muscular strength has been linked to less pain, fewer difficulties in completing daily activities, and improved mobility [43], which was confirmed in our results for both groups, supporting that IMS is an effective modality.

Overall, limitations are identified in addition to the relevant results found. The study was limited to women affected by meniscal lesions and whose results cannot be generalized for all individuals with meniscal lesions. In addition, during the first three weeks of the protocol, muscle soreness was felt by some patients who could not bear it, caused inevitably by the short recovery time between sets from the second training week. This prompted them to consider the extensive effort required for the program. Moreover, the number of sessions/week was limited due to the availability of the isokinetic dynamometer. Additionally, the knee testing order during isokinetic procedures was not randomized due to the times of availability and convenience of patients during the experimental protocol evaluations. Finally, examiner intra-rater reliability could not be provided and calculated due to the lack of time that was allocated to use the experimental area and equipment.

Our results are preliminary, and it seems that future studies are necessary that involve a larger number of participants and a longer training duration. A male population and a comparison between surgical procedures and the IMS program should also be possible.

## 5. Conclusions

Our findings support the isokinetic muscle strengthening (IMS) program in the active mode as an effective therapeutic modality in managing middle-aged patients suffering from a degenerative meniscal tear. We found a significant improvement in physical performance, range of motion, life quality and relief in knee pain. In addition, no significant difference in concentric knee flexor or extensor (body weight adjusted) strength between groups at velocities of 60°/s and 180°/s was noted, which can be explained by the rather short duration of the intervention program (6 weeks). Structures and functional rehabilitation centers in physical medicine with isokinetic dynamometers should introduce IMS programs for the better management of this type of pathology, making the rehabilitation process more effective than the classic one proposed.

## Figures and Tables

**Table 1:** Extensor and flexor knee peak torque pre- and post-intervention in concentric mode at 60°/s and 180°/s.

			Con 60°/s		Con 180°/s	
			EPT	FPT	EPT	FPT
**EG**	**R**	**Pré**	1.02 ± 0.38	0.54 ± 0.25	0.47 ± 0.19 ^α^	0.28 ± 0.11
		**Post**	1.04 ± 0.37	0.60 ± 0.28 ^+^	0.55 ± 0.19 ^+^	0.32 ± 0.12
	**L**	**Pré**	1.00 ± 0.33	0.56 ± 0.15 *	0.38 ± 0.22	0.27 ± 0.07
		**Post**	1.22 ± 0.48 *^,+^	0.55 ± 0.15	0.44 ± 0.21	0.29 ± 0.08
**CG**	**R**	**Pré**	0.88 ± 0.39	0.48 ± 0.15	0.40 ± 0.21	0.28 ± 0.09 ^+^
		**Post**	1.11 ± 0.37 *	0.53 ± 0.25	0.63 ± 0.32 *^,+^	0.42 ± 0.17 *^,++^
	**L**	**Pré**	0.90 ± 0.30	0.38 ± 0.11	0.40 ± 0.18	0.21 ± 0.07
		**Post**	0.98 ± 0.32	0.56 ± 0.28 **	0.53 ± 0.21 *^,+^	0.26 ± 0.08 ^α^

Note: Data are reported as mean ± standard deviation. EG: experimental group; CG: control group; Pre: pre-program; Post: post-program; R: right side; L: left side; Con: concentric; EPT: extensor peak torque; EPT: flexor peak torque; * *p* < 0.05: Pre vs. Post; ^+^ *p* < 0.05: R/L vs. Pre/Post; ** *p* < 0.05: EG vs. CG; ^++^ *p* < 0.05: EG/CG vs. Pre/Post; ^α^ *p* < 0.05: R/L vs. EG/CG.

**Table 2:** Physical performance pre- and post-program.

	Sit and Stand Test		Stair Climb Test	
	Pre	Post	Pre	Post
**EG**	9.67 ± 1.50	19.08 ± 1.68 ***^,++^	7.26 ± 1.16	5.13 ± 1.04 ***^,+^
**CG**	9.50 ± 1.51	14.00 ± 3.74 ***	7.30 ± 0.56	6.69 ± 1.07

Note: data are reported as mean ± standard deviation. EG: experimental group; CG: control group; Pre: pre-program; Post: post-program. *** *p* < 0.001: Pre vs. Post; ^+^ *p* < 0.05: EG vs. CG; ^++^ *p* < 0.001: EG vs. CG.

**Table 3:** Knee amplitude test (degree) in flexion and extension for both sides.

		Flex-R	Flex-L	Ext-R	Ext-L
**EG**	**Pre**	121.25 ± 10.03	122.92 ± 8.65	5.42 ± 7.82	3.75 ± 5.69
	**Post**	133.75 ± 7.11 ***	134.58 ± 5.82 ***	2.08 ± 3.34	2.08 ± 3.34
**CG**	**Pre**	123.75 ± 6.44	125.83 ± 6.34	7.08 ± 8.11	7.08 ± 8.11
	**Post**	134.67 ± 5.02 ***	135.08 ± 5.07 ***	3.75 ± 4.33	3.33 ± 4.44 ***

Note: data are reported as mean ± standard deviation. EG: experimental group; CG: control group; Pre: pre-program; Post: post-program; Flex-R: right knee flexion; Flex-L: left knee flexion; Ext-R: right knee extension; Ext-L: left knee extension. *** *p* < 0.001: Pre vs. Post.

**Table 4:** Knee amplitude test (degree) in flexion and extension for both sides.

		Heel-to-Buttock Distance		Pain in Affected Knee (Thessaly Test)	
		R	L	Rotation R	Rotation L
**EG**	**Pre**	39.50 ± 5.63	38.75 ± 4.97	7.42 ± 1.08	7.42 ± 1.00
	**Post**	33.08 ± 5.78	33.08 ± 5.12	6.42 ± 1.08	6.00 ± 1.04 ***^,+^
**CG**	**Pre**	38.58 ± 4.50	38.08 ± 4.12	6.92 ± 1.24	6.92 ± 0.51
	**Post**	33.92 ± 6.60	33.42 ± 6.57	5.92 ± 1.24 ^+^	5.67 ± 0.65 ***^,++^

Note: data are reported as mean ± standard deviation. EG: experimental group; CG: control group; Pre: pre-program; Post: post-program; L: left; R: right. *** *p* < 0.001: Pre vs. Post; ^+^ *p* < 0.05: Rotation R vs. Rotation L and Pre vs. Post; ^++^ *p* < 0.001: EG vs. CG and Rotation R vs. Rotation L.

**Table 5:** Program’s effect on the quality of life and pain through the KOOS questionnaire.

	KOOS-Pain		KOOS-QOL	
	Pre	Post	Pre	Post
**E.G**	27.42 ± 4.23	4.00 ± 1.86 ***	13.25 ± 1.36	2.92 ± 1.73 ***
**C.G**	28.83 ± 4.09	8.83 ± 11.36 ***	13.67 ± 1.78	4.58 ± 3.23 ***

Note: data are reported as mean ± standard deviation. EG: experimental group; CG: control group; QOL: quality of life. *** *p* < 0.001: Pre vs. Post.

## Data Availability

Data are available upon request from the corresponding author.
